# 3D time series analysis of cell shape using Laplacian approaches

**DOI:** 10.1186/1471-2105-14-296

**Published:** 2013-10-04

**Authors:** Cheng-Jin Du, Phillip T Hawkins, Len R Stephens, Till Bretschneider

**Affiliations:** 1 Warwick Systems Biology Centre, University of Warwick, Coventry CV4 7AL, UK; 2 The Babraham Institute, Cambridge CB22 3AT, UK

## Abstract

**Background:**

Fundamental cellular processes such as cell movement, division or food uptake critically depend on cells being able to change shape. Fast acquisition of three-dimensional image time series has now become possible, but we lack efficient tools for analysing shape deformations in order to understand the real three-dimensional nature of shape changes.

**Results:**

We present a framework for 3D+time cell shape analysis. The main contribution is three-fold: First, we develop a fast, automatic random walker method for cell segmentation. Second, a novel topology fixing method is proposed to fix segmented binary volumes without spherical topology. Third, we show that algorithms used for each individual step of the analysis pipeline (cell segmentation, topology fixing, spherical parameterization, and shape representation) are closely related to the Laplacian operator. The framework is applied to the shape analysis of neutrophil cells.

**Conclusions:**

The method we propose for cell segmentation is faster than the traditional random walker method or the level set method, and performs better on 3D time-series of neutrophil cells, which are comparatively noisy as stacks have to be acquired fast enough to account for cell motion. Our method for topology fixing outperforms the tools provided by SPHARM-MAT and SPHARM-PDM in terms of their successful fixing rates. The different tasks in the presented pipeline for 3D+time shape analysis of cells can be solved using Laplacian approaches, opening the possibility of eventually combining individual steps in order to speed up computations.

## Background

Cell migration is a highly complex process that integrates many spatial and temporal cellular events [1]. It plays important roles in embryonic development, tissue repair, cancer invasion and atherosclerosis [2]. Recent advances in live-cell imaging yield vast amounts of image data [3], and a number of image analysis algorithms with high throughput capability have been developed [4,5]. These were applied for example to characterize mutants that lack the ability to sense gradients of a chemoattractant, or contract their cell body less efficiently while moving.

Our current view of moving cells is mostly based on 2D cross-sections through the centre of cells or evanescent wave imaging of the substrate attached cell surface. Similarly, software developed for cell migration studies focuses primarily on migration in 2D. Although treating cells as 2D entities has proven effective in understanding some aspects of cell locomotion and in identifying defects in a variety of mutants [6], neglecting the third dimension [7] results in several misconceptions [6]. Two-dimensional cross-sections give the wrong impression of cells being flat and uniformly attached, which in first approximation is adopted in many models of cell polarity and organization, although it is clear that the differences between the front and rear of a cell are as big as those between the ventral and dorsal sides. Secondly, we falsely tend to assume that small shape changes in 2D cross-sections are accompanied by similarly small changes in the third dimension. Thirdly, we often ignore that *in vivo* cells may crawl through complex 3D environments which can dramatically change cell behavior and the way that cells polarize when compared to 2D movement in a dish [7].

Recent advances in live cell microscopy have made it possible to acquire high quality 3D+time volumetric images of cell migration. Currently, the most widely applied 3D fluorescence imaging technique is fast spinning disk confocal microscopy which can typically acquire a stack of 30 slices within a few seconds and is therefore capable of imaging cellular deformations on the second timescale [8]. Since large and complex data sets typically consist of 5,000–10,000 single images [9], analysis tools with high throughput capability are needed.

Although cell images can be visualized by methods of volume and surface rendering, both lack descriptive power. Ideally we want to characterize global and local shape features by a manageable number of parameters. A concise description should allow for accurate comparison of object shapes in order to find dissimilarities, and for matching objects to predefined models, as well as for efficient reconstruction and manipulation of objects [10]. The ultimate goal is to develop automated, efficient and objective methods that can create spatio-temporal maps of signaling transduction and corresponding cell surface deformations in order to further our functional understanding of cell motility in a quantitative way.

The most advanced software for analysing cell shape and motility of amoeboid cells such as neutrophils or *Dictyostelium* is 3D-DIAS [11], which is commercially available. Models of cell surfaces are mathematically reconstructed by beta-spline functions. 3D-DIAS allows visualization of 3D dynamics of cell surfaces, but since it works with lower contrast DIC images and not fluorescence the resolution of the generated surface models is not optimal. Also, it lacks automated analysis of cell surface deformations and fluorescence. Current commercial software for quantifying 3D fluorescence images include Meta-Morph (Molecular Devices, Sunnyvale, USA), Volocity (Perkin-Elmer, Waltham, USA) and Imaris (Andor Technology, Belfast, Northern Ireland), but they offer little in terms of cell shape analysis. Advanced software has recently become available for analyzing simple cell shape changes of plants in 3D [12].

Inspired by the previous works of [4] and [11], we present a new framework for 3D shape analysis of highly dynamic cells. In [4], 2D time-lapse images of moving cells were mapped onto the unit disk, which served as a reference frame both for registering cells across time and comparing different cells with varying shapes. Here, we use spherical parameterization to map cell surfaces onto the unit sphere. Spherical parameterization has been used extensively for brain cortex shape analysis [13,14]. Several open source software toolboxes are available such as SPHARM-PDM (Point Distribution Models) [15] and SPHARM-MAT (Modeling and Analysis Toolkit) [16]. Since brain images from different time points or even from different individuals are quite similar, a normalized template is often used for shape analysis. In our application, however, cell shapes of moving cells usually vary greatly between different time points, and there is no common template. Although spinning disk confocal microscopy allows acquiring time series of 3D stacks within seconds, short exposure times result in low signal-to-noise ratios. Further complications can be inhomogeneous labelling (e.g. the intensity of the cell interior is similar to that of the background), and ambiguous boundaries, making segmentation non-trivial.

**Figure 1:**
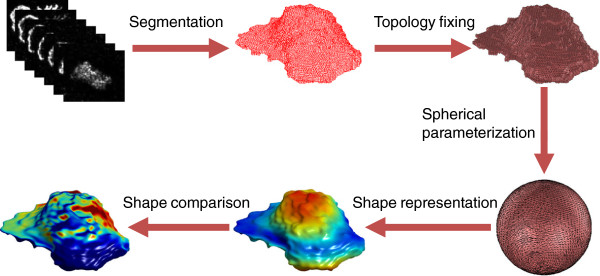
**The workflow of the proposed approach includes five major steps: cell segmentation, topology fixing, spherical parameterization, shape representation, and shape comparison.** Quantitative analyses of cell shape firstly require segmentation of individual cells. The topology of the segmentation might need fixing to create a simply connected 3D object, a requirement for the subsequent step of spherical parameterization. Based on the topology fixed binary volume and the spherical parameterization result, shape representation techniques such as spherical harmonics are employed to describe cell shapes. After that, measurements of local and global dynamic cell shape changes can be conducted.

Our proposed framework includes five major steps: cell segmentation, topology fixing, spherical parameterization, shape representation, and shape comparison as illustrated in Figure 1. In this work, we will mainly focus on the first four steps while only briefly describing the final step for completeness. We extend our work for segmentation of individual cells [17] by adding an automatic seed detection method. A novel topology fixing method is then developed to create simply connected 3D objects required for spherical parameterization. Both cell segmentation and topology fixing are formulated using a Laplacian approach. We use the spherical parameterization method proposed in [10], and represent cell shape with spherical harmonics (SPHARM), which are very popular methods in brain cortex shape analysis. The techniques used for spherical parameterization and shape representation are again closely related to the Laplacian operator. Based on the above four steps, measurements of cell membrane deformations can be finally conducted. Historically, a variety of solutions have been proposed for each of the above-mentioned steps. We here employ a common Laplacian approach. Although currently we are unable to exploit this feature to connect the different steps in an operative manner, we believe that a unified approach will help working towards this goal in future.

## Methods

### Segmentation

The segmentation of volumetric cell scans yields a partitioning in the form of binary 3D stacks. We begin by briefly explaining notations. Given an image with *N* voxels, a weighted undirected graph *G*^*S*^ = (*V*, *E*, *A*^*S*^) with vertices *v* ∈ *V* and edges *e* ∈ *E* is constructed. Each voxel corresponds to a vertex *v*, and each pair of neighbouring vertices is connected by an edge *e*. The connectivity is 6 or 26 for 3D images. *A*^*S*^ ∈ *R*^*N*×*N*^ is a weighted adjacency matrix, which maps changes in the image structure to edge weights. The weighting function is defined as

(1) $$A_{\text{ij}}^{s}=\exp\left(-{ǁS_{i}-S_{j}ǁ}^{2}/\sigma_{S}-{ǁI_{i}-I_{j}ǁ}^{2}/\sigma_{I}\right)$$

where *I*_*i*_ is the image intensity value at vertex *v*_*i*_, and *S*_*i*_ indicates its spatial position. The graph Laplacian matrix is then given by *L*^*s*^ = *D*^*s*^ - *A*^*s*^ where *D*^*s*^ is a diagonal matrix with elements given by the degree of vertices $d_{i}^{s}=\sum_{j\in V}A_{\text{ij}}^{s}$.

Care should be taken if the voxel spacing in z is different from that in x and y, and any anisotropy should be corrected by appropriate scaling factors. As we work with images which are downsampled in x and y (as detailed below) voxel spacings in x, y and z are almost identical and no correction is applied. When imaging dynamic processes, voxels within a given z-plane are theoretically more likely to be correlated since planes are acquired sequentially with some delay. In principle this could result in higher affinity values within a single plane, but as it is unclear how to quantify this effect we ignore it for now.

It is worth to note that the graph Laplacian matrix *L* plays a key role in our framework. By building problem specific *L*, such as *L*^*s*^ the Laplacian matrix for segmentation, and exploring its intrinsic eigenstructures, not only segmentation but also the subsequent steps including topology fixing, spherical parameterization, and shape representation can be performed.

#### Random walker method

The random walker method proposed in [18] is a semi-automatic method, which requires the user to provide seeds indicating foreground or background. It has many nice characteristics, including trivial generalization to simultaneous multi-region and 3D segmentation, robustness to noise, weak boundaries detection, a sound theoretical basis, and a probabilistic output that can be employed to identify areas of uncertainty. The probabilities of unseeded nodes are found by

(2) $$\underset{x}{\text{min}}\;x^{T}L^{s}x=\underset{e_{\text{ij}}\in E}{\sum }A_{\text{ij}}^{s}{\left(x_{i}-x_{j}\right)}^{2}$$

where *x*_*i*_ represents the probability of pixel *i* being foreground or background. Based on the provided seeds, Eq. (2) can be decomposed into

(3) $$\underset{x}{\text{min}}\;\left[x_{M}^{T}\;x_{U}^{T}\right]\;\left[\begin{array}{cc} L_{M}^{s} & B\\ B^{T} & L_{U}^{s} \end{array}\right]\;\left[\begin{array}{c} x_{M}\\ x_{U} \end{array}\right]$$

where the nodes in *L*^*s*^ and *x* are ordered such that seed nodes come first and unseeded nodes second, *x*_*M*_ and *x*_*U*_ correspond to the probabilities of seeded and unseeded nodes respectively, *B* is a submatrix of *L*^*s*^. The optimization problem of Eq. (3) can be solved by the following linear equation:

(4) $$L_{U}^{s}x_{U}=-B^{T}x_{M}$$

This is a large linear system for 3D segmentation of a cell image. Although Eq. (4) is a sparse, symmetric and diagonally dominant system, solving it directly will still be slow requiring *O*(*U*) running time, *U* representing the total number of unseeded nodes.

#### Downsampling

Obviously, if we reduce the number of unseeded nodes, the time for solving Eq. (4) can be reduced. The problem is how to reduce the number of equations without sacrificing accuracy. We first downsample the image using the bilinear method to make it substantially smaller. Since the resolution in z is much lower than in x and y, we do not downsample in z. The random walker method is then applied to the downsampled stack, with many fewer equations to be solved.

#### Automatic seeds detection

Both the original random walk [18] and our previous method [17] require manual seeds for segmentation. Here we propose an automatic seeding method, which is inspired by the recent advances on saliency detection via Fourier domain analysis [19,20]. Working in the frequency domain is simple and fast, but more importantly these methods usually involve downsampling the image which is in line with our segmentation approach.

The downsampled image *I*_*d*_ is firstly transformed into the Fourier domain *F*(*I*_*d*_). Amplitude *A*_*I*_(*F*(*I*_*d*_)) = |*F*(*I*_*d*_)| and phase *P*(*F*(*I*_*d*_)) = *angle*(*F*(*I*_*d*_)) spectra are then calculated. The log spectrum is obtained by

(5) $$\text{LS}\left(I_{d}\right)=\log A_{I}\left(F\left(I_{d}\right)\right)$$

A Gaussian kernel *h* is used to smooth out the spikes in the log spectrum

(6) $$A_{s}\left(F\left(I_{d}\right)\right)=\text{LS}\left(I_{d}\right)*h$$

The saliency map of the downsampled image *I*_*d*_ can then be computed as

(7) $$S\left(I_{d}\right)={\left(F^{-1}\left(\exp\left(A_{s}\left(F\left(I_{d}\right)\right)+\text{iP}\left(F\left(I_{d}\right)\right)\right)\right)\right)}^{2}*h$$

where the smoothed log spectrum in Eq. (6) and the original phase spectrum are combined to compute the inverse Fourier transform.

The motivation behind the above approach is that if we divide the image into small patches, the majority of them will consist of background with similar patterns, which show up as spikes in the log spectrum after transforming the image into the frequency domain [19]. These spikes, and with it the background, are suppressed by applying a low-pass Gaussian filter.

Based on the saliency map obtained in Eq. (7), a simple two-level threshold method is proposed to detect foreground and background seeds, respectively. The first level threshold values for foreground and background are calculated as

(8) $$T_{f1}=\text{mean}\left(S\left(I_{d}\right)\right)+2\text{std}\left(S\left(I_{d}\right)\right)$$

(9) $$T_{b1}=\text{mean}\left(S\left(I_{d}\right)\right)+\text{std}\left(S\left(I_{d}\right)\right)$$

The first level foreground and background are obtained below

(10) $$F_{1}\left(i\right)=\left\{\begin{array}{ccc} I_{d}\left(i\right) & \;\mathrm{if}\; & S\left(i\right)>T_{f1}\\ 0 & & \text{otherwise} \end{array}\right)$$

(11) $$B_{1}\left(i\right)=\left\{\begin{array}{ccc} I_{d}\left(i\right) & \;\mathrm{if}\; & S\left(i\right)<T_{b1}\\ 0 & & \text{otherwise} \end{array}\right)$$

The second level threshold values for the foreground and background are

(12) $$T_{f2}=\text{mean}\left(F_{1}\left(i\right)\right)$$

(13) $$T_{b2}=\text{mean}\left(B_{1}\left(i\right)\right)+2\text{std}\left(B_{1}\left(i\right)\right)$$

The second level foreground and background are computed via

(14) $$F_{2}\left(i\right)=\left\{\begin{array}{lr} 1 & \;\mathrm{if}\;I_{d}\left(i\right)>T_{f2}\\ 0 & \text{otherwise} \end{array}\right)$$

(15) $$B_{2}\left(i\right)=\left\{\begin{array}{lr} 1 & \;\text{if}\;I_{d}\left(i\right)<T_{b2}\\ 0 & \text{otherwise} \end{array}\right)$$

which are used as the input of seeds for the cell segmentation in Eq. (4).

The above method for seed detection works well for cell images with only one stain. We threshold the saliency map by estimating an initial foreground and background as deviations from the mean intensity and then refine these estimates. Instead of using a single threshold value for the segmentation, we use two clearly separated threshold values, one for foreground and the other for background to be on the safe side. This is used as input for a more accurate segmentation employing the random walker method. The threshold selection is not sensitive in our algorithm. Using Otsu’s method [21] for the initial estimation of foreground and background of Eqs. (10 and 11) similar results were obtained.

The proposed method has also been applied to a different cell type (*Dictyostelium* cell with LimE tagged by mRFP, and Coronin fused to GFP, combining two channels in one, see Additional file 1: Figure S1, volume size: 389 × 292 × 73). In this case we need to slightly tune the second level threshold values of Eqs. (12 and 13) as the standard deviation of the combined two channel images is much bigger than that of the single labelled neutrophil images.

#### Edge-preserved upsampling

The random walker method of Eq. (4) is applied to the downsampled image *I*_*d*_ with automatically detected seeds. The obtained downsampled probability is extrapolated by a cubic method to the original image size. The upsampled probability $q_{i}^{c}$ at pixel *i* is calculated in the following form:

(16) $$q_{i}^{c}=\frac{1}{\left|\text{neighb}\left(i\right)\right|+1}\sum_{k\in \left\{\text{neighb}\left(i\right),i\right\}}w\left(I_{i},\mu_{\text{seed}}^{c}\right)\left(p_{i}^{c}+\eta \right)$$

where *c* is the class indication of cell or background, *I*_*i*_ is the intensity at pixel *i* of the original image, $\mu_{\text{seed}}^{c}$ is the mean intensity of seeds, $p_{i}^{c}$ is the extrapolated probability of class *c* at pixel *i*, and *η* is a scalar parameter to balance the importance of the weight $w\left(I_{i},\mu_{\text{seed}}^{c}\right)$ given by

(17) $$w\left(I_{i},\mu_{\text{seed}}^{c}\right)=\left\{\begin{array}{cc} {\left(1-{\left(\left(I_{i}-\mu_{\text{seed}}^{c}\right)/\tau \right)}^{2}\right)}^{2} & \left|I_{i}-\mu_{\text{seed}}^{c}\right|\leq \tau \\ 0 & \text{otherwise} \end{array}\right)$$

where *τ* is a scale parameter in the intensity domain to control the influence of *I*_*i*_. Eq. (17) gives higher weight to pixels with intensities similar to the mean intensity of seeds. If the intensity difference is bigger than *τ*, the weight is set to zero. The final segmentation is obtained by assigning the pixel *i* to the class corresponding to $\max_{c}\left(q_{i}^{c}\right)$.

The effect of Eq. (17) is similar to Tukey’s biweight edge-stopping function introduced in [22] for robust anisotropic diffusion. The discrete diffusion equation governing the value $I_{i}^{t+1}$ is

(18) $$I_{i}^{t+1}=I_{i}^{t}+\frac{\alpha }{\left|\text{neighb}\left(i\right)\right|}\underset{k\in \text{neighb}\left(i\right)}{\sum }g\left(I_{k}^{t}-I_{i}^{t}\right)\left(I_{k}^{t}-I_{i}^{t}\right)$$

where *t* is the time step, the constant *α* is a scalar value that determines the rate of diffusion, and *g*(∙) is an edge-stopping function. The choice of *g*(∙) can greatly affect the extent to which discontinuities are preserved. A variety of edge-stopping functions have been used such as Lorentz, Gauss, and Tukey’s biweight function [22], which are plotted in Additional file 2: Figure S2. It is helpful to understand how an edge-stopping function deals with outliers. We can see that the Lorentz function gives more influence to outliers than the Gauss and the Tukey functions. More robust results can be achieved by Tukey’s biweight function, as it completely prevents diffusion across edges.

In [22], the intensity difference is calculated between the pixel *i* and its neighbouring pixels as seen in Eq. (18). For regions of piecewise constant intensity, anisotropic diffusion is equivalent to averaging intensities of neighbour pixels. For an image region that includes boundaries, the influence of "outliers" is low as the value of *g*(∙) is small for large intensity differences. However, our method computes intensity differences between pixel *i* and the mean of seeded pixels, instead of its neighbouring pixels. To make it more clear, Eq. (16) can be rewritten as

(19) $$q_{i}^{c}=\frac{1}{\left(\left|\text{neighb}\left(i\right)\right|+1\right)\left|\text{see}d^{c}\right|}\underset{k\in \left\{\text{neighb}\left(i\right),i\right\}}{\sum }\underset{j\in \mathrm{see}d^{c}}{\sum }w\left(I_{i},I_{j}^{c}\right)\left(p_{i}^{c}+\eta \right)$$

where |*seed*^*c*^| is the cardinality of the set *seed*^*c*^, the seeds of class *c*. Here, the edge-stopping property is nicely kept, and "outliers" can be explained explicitly as pixels belonging to a class other than *c*.

The final segmentation result will contain some small spurious objects due to a few high intensity pixels in the background. The morphological close operator can be used to remove those falsely detected small objects.

### Topology fixing

The subsequent step of spherical parameterization requires cell surfaces to have spherical topology, i.e. a genus zero surface [23], the value of a surface’s genus is equal to the number of "holes" it has. The surface of an ideal cell can be considered as genus zero. Limitations in practice are that cells often exhibit thin protrusions, which can cross, or fluorescent markers are not distributed evenly, each of which can result in holes in the segmentation.

The two open-source SPHARM softwares, SPHARM-PDM [15] and SPHARM-MAT [16] provide tools for fixing the topology of 3D binary objects. Our results show however that these tools fail to fix the topology of cells with complex protrusions. We also tried morphological operators such as dilation and close, which can fix topology to some extent, but at the expense of altering the original binary volume significantly. All the methods mentioned above fix topology based on binary volumes.

Here we present a new method for fixing cell topology which minimizes aliasing artefacts. Instead of operating on the binary volume directly, we treat it as a set of hard constraints imposed on the separating surface that encompasses the centres of all foreground voxels. A non-binary underlying embedding function is obtained by solving a constrained convex optimization problem. Based on the embedding function, protrusions are detected and those causing ill topology are identified. The identified ill protrusions are then fixed, while leaving other parts of the volume unaffected. Such a strategy has several advantages over using the binary volume directly, including the ease of fixing topology as well as minimizing aliasing artefacts.

#### Non-binary embedding

We are looking for a continuous embedding function, the zero-isosurface which is compatible with the original binary volume. There are many choices of the embedding that meet the hard constraints imposed by the binary volume. The work of [24] took a constrained area minimization approach in the implicit level set framework. Lempitsky (2010) [25] suggested a method that operates within the implicit framework as well and minimized a higher-order smoothness criterion imposed on the embedding function. Here we follow the idea of imposing the first-order smoothness on the embedding function for image segmentation as outlined in the last section. Given a binary volume, we build a graph *G*^*t*^ from the discrete grid domain of the binary image. A graph Laplacian matrix *L*^*t*^ of *G*^*t*^ is constructed in a similar way to the last section. However, each element $A_{\text{ij}}^{t}$ of the adjacency matrix *A*^*t*^ of the graph *G*^*t*^ is computed differently as

(20) $$A_{\text{ij}}^{t}=\left\{\begin{array}{c} 1\;\mathrm{if}\;v_{i}v_{j}\in E\\ 0\;\text{otherwise} \end{array}\right)$$

### First-order embedding

A general optimization-based regularization formulation for finding the embedding function *F* with first-order smoothness minimizes the following p-Dirichlet integral functional

(21) $$\min\int_{\text{Ω}}\frac{1}{p}{\left|\nabla F\right|}^{p}d\Omega \;s.t.v_{\text{ijk}}f_{\text{ijk}}\geq 0$$

where *f*_*ijk*_ ∈ *R* denotes the value of *F* on each vertex of the graph. The value on each vertex *v*_*ijk*_ is +1 if it belongs to foreground, otherwise -1. Minimizing the integral of the p-power of the variation forces the embedding function to remain smooth. The set of hard constraints enforces its fidelity, so that the function is compatible with the original binary volume. The different values of *p* lead to different formulations for optimizing the energy of Eq. (21).

For p=1, Eq. (21) becomes

(22) $$\min\int_{\Omega }\left|\nabla F\right|d\Omega \;s.t.v_{\text{ijk}}f_{\text{ijk}}\geq 0$$

where $\left|\nabla F\right|=\sqrt{{\left(\partial F/\partial x\right)}^{2}+{\left(\partial F/\partial y\right)}^{2}+{\left(\partial F/\partial z\right)}^{2}}$ is the *L*_l_ norm of the gradient magnitudes. Eq. (22) corresponds to the total variation minimization of the embedding function *F* in a domain *Ω*. In this case, the area of the zero-isosurface is minimized, leading to "shrinking bias", i.e. bias towards smaller surfaces [25]. Eventually, the optimizer converges to an empty surface, which is the unique global solution of such regularization.

If we substitute p=2 into Eq. (21), it minimizes the Dirichlet integral defined as

(23) $$\min\frac{1}{2}\int_{\text{Ω}}{\left|\nabla F\right|}^{2}d\Omega \;s.t.v_{\text{ijk}}f_{\text{ijk}}\geq 0$$

A combinatorial formulation of Eq. (23) using the graph Laplacian matrix *L*^*t*^ has the following form

(24) $$\min\frac{1}{2}f^{T}L^{t}f\;s.t.v_{\text{ijk}}f_{\text{ijk}}\geq 0$$

The regularization and the hard constraints of Eq. (24) would lead to a trivial optimal solution of *F* ≡ 0. We use a similar method as in [25] where we add a margin *m*_*ijk*_ to separate the resulting embedding function from the zero solution. Thus, Eq. (24) changes to

(25) $$\min\frac{1}{2}f^{T}L^{t}f\;s.t.v_{\text{ijk}}f_{\text{ijk}}\geq m_{\text{ijk}}$$

where the margin *m*_*ijk*_ is calculated as the Euclidean distance to the set *B* of boundary nodes in *V*:

(26) $$m_{\text{ijk}}=\underset{\left(x,y,z\right)\in B}{\text{min}}\sqrt{{\left(i-x\right)}^{2}+{\left(j-y\right)}^{2}+{\left(k-z\right)}^{2}}$$

### Implementation

To solve the convex quadratic problem of Eq. (25) we applied a simple optimization scheme based on projected Jacobi iterations similar to [26]. The matrix *L*^*t*^ is factorized into the diagonal part *D*^*t*^, where $D_{\text{ii}}^{t}=L_{\text{ii}}^{t}$, and $D_{\text{ij}}^{t}=0$ for *i* ≠ *j*, and the off-diagonal part *C*^*t*^ = *D*^*t*^ - *L*^*t*^ with zero diagonals. The iterations are based on the following formula:

(27a) $$f_{\text{Jacobi}}^{k+1}=-{\left(D^{t}\right)}^{-1}C^{t}f^{k}$$

(27b) $$\;f_{\text{step}}^{k+1}=\lambda \cdot f_{\text{Jacobi}}^{k+1}+\left(1-\lambda \right)f^{k}$$

(27c) $$\;f^{k+1}=\min\left\{h,\max\left\{l,f_{\text{step}}^{k+1}\right\}\right\}$$

where the scalar *λ* is the step length set to 0.5 in our experiments, parameters *l* and *h* are the lower and higher bounds on the values of *f* derived from the margin inequality that are taken element-wise. To speed up the algorithm, we define a narrow band *S* of vertices obtained by thresholding the margin *m*_*ijk*_:

(28) $$\left\{\left(i,j,k\right)\left|m_{\text{ijk}}<t\right)\right\}$$

where the threshold *t* is the half-width of the band. The computation is confined to the narrow band *S*, i.e. only those vertices near the surface within a certain distance are considered instead of performing the computation on the full grid.

#### Fixing ill topology

The goal of the non-binary embedding is to estimate a continuous function from binary segmented data that retains fine details presented in the original segmentation. To fix any ill topology, we suggest to proceed in a reverse manner with respect to the non-binary embedding. We treat the binary volume as a threshold of a discrete sampling of the obtained embedding function *F*, and recover it in a divide and conquer approach. Here, we first propose a method for protrusion detection based on the embedding function *F*, and then those protrusions that cause the ill topology are fixed.

### Protrusion detection

The assumption we make here is that ill topologies are caused by cellular protrusions. We observe cells that undergo spreading, which mean they are initially spherical when attaching to a coverslip, and correspondingly the segmented binary volumes have a well defined spherical topology. However, when cells start adhering more firmly to the substrate and start migrating they develop thin protrusions, more precisely filopodia and retraction fibres. These often create holes when crossing each other so that topology fixing is required before one can proceed with further processing, such as spherical parameterization.

**Figure 2:**
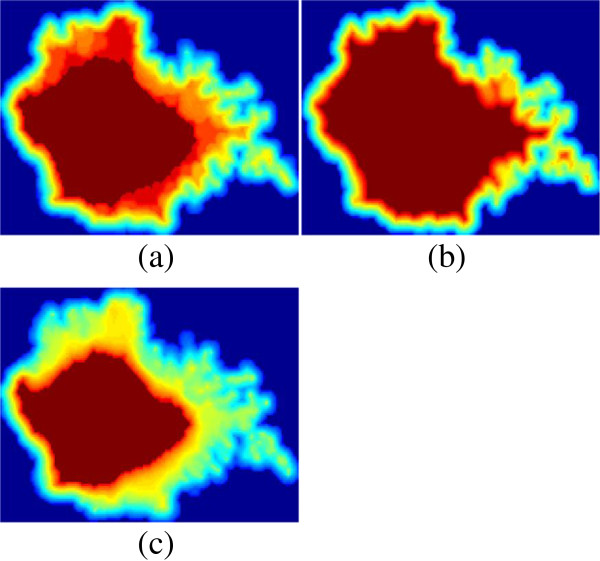
**Non-binary embedding of a 3D stack of a neutrophil cell. (a**-**c)** are the 5th, 8th, and 11th slices. The estimated embedding function is in the form of a level set of a grey-scale volume. This representation facilitates detecting or filling-in of holes. Topology is evaluated starting from inside (levels with high value) and progressing towards the outer layers (low values) to find the largest thresholded binary volume with spherical topology.

The embedding function *F* we obtained is similar to that in [24], where the constrained minimal area optimization problem is solved in the implicit level-set framework. As seen in Figure 2, the estimated embedding function *F* is in the form of a level set of a grey-scale volume. The benefit of interpreting the embedded function as level-set implicit representation is the elegant handling of changes in topology such as detecting or filling-in of holes. Thus, we are able to evaluate the topology of a given cell from inner layers (levels with high value) to outer layers (levels with low value). The basic idea is to march through the level sets and progress towards a desired one whose thresholded binary volume has a spherical topology.

We start initially outside, with low level sets, where *F* is thresholded with a value of 1.5 yielding a binary volume. To ensure it has spherical topology, its topology is evaluated by computing the Euler-Poincaré characteristic. If not, we increase the threshold and repeat the above procedure. We obtain a binary volume *A* that can be considered as a shrunk version of the original volume. After that we gradually enlarge the surfaces of the binary volume *A* via simple morphological dilation. The size of the structuring element is related to the narrow band width *S* as defined in Eq. (28), initially set as (t + 1) × (t + 1) × (t + 1), and could be changed adaptively. Protrusions are detected by simply subtracting the dilated binary volume from the original one. We only keep subtracted results with values higher than 0 to make it consistent with the original binary volume. Besides protrusions, we also obtain the remaining binary volume *B*, which consists of the main cell body.

The above procedure in some ways mimics low-pass filtering, such as Gaussian filtering. The volume *B* can be considered as low frequency content, while protrusions represent high frequencies. However, our method is fundamentally different from the Gaussian filtering approach. It is unknown how to choose the kernel size for the Gaussian filtering approach, so that the filtered binary volume has spherical topology. Furthermore, since the filtered result is incompatible with the original binary volume, it is unlikely that the fine details smoothed out by the filtration contain all portions that cause ill topology.

### Fixing ill protrusions

In general, only a few protrusions contribute to ill topologies. We evaluate them one by one by adding them back to the binary volume *B*. If a protrusion does not violate the genus zero topology requirement, we merge it back into the binary volume *B*. In this way, we identify those protrusions that cause the topology change and a binary volume *C* that is the combination of the binary volume *B* and those protrusions with spherical topology.

After the identification of ill protrusions, we treat each individual one as a single binary volume. Again, we embed each protrusion into a non-binary function using the method detailed before. Thanks to the level set form of the obtained embedding function it is easy to fix the topology. We simply expand each protrusion by using a low level set, -0.1-sets to start with. We check the topology of the expanded binary volume, and decrease the threshold value if the binary volume still violates spherical topology.

As protrusions are usually very small compared to the original volume, the fixing procedure is very efficient. Once protrusions are fixed, we merge them back to the binary volume *C*. Finally, we obtain a binary volume with spherical topology, where only a small portion containing fixed protrusions has been modified, while the majority of the binary volume is unchanged.

### Spherical parameterization

The objective of spherical parameterization is to embed the cell surface in the unit sphere while minimizing the distortion of the surface net in the mapping. Generally, a good mapping attempts to either minimize length, angle, or area distortions. For comparisons between cell shapes acquired at sufficiently close enough time points we assume the total cell surface area to change only minimally, and consider an equiareal mapping as justifiable: Here, a particular region on one cell surface is always compared with the corresponding region at a subsequent point in time, where both regions have the same area [27].

Spherical parameterization creates a continuous, uniform mapping from the cell surface onto the unit sphere. The result is a bijective mapping between each point **p** on the cell surface *M* and a pair of spherical coordinates:

(29) $$p\left(\theta ,\varphi \right)={\left(x\left(\theta ,\varphi \right),y\left(\theta ,\varphi \right),z\left(\theta ,\varphi \right)\right)}^{T}$$

where the polar angle *θ* ∈ [0,*π*] is the angle between the positive z-axis (north pole) and the vector corresponding to **p**. *φ* ∈ [0,2*π*] is the azimuthal angle between the positive x-axis and the projection of **p** onto the x-y plane. We have tried to use several different spherical parameterization methods [10,27-29], among them the method presented in [10] turned out to perform best for our application. Here we briefly describe the key idea behind the algorithm and highlight that the method is closely related to the graph Laplacian matrix *L*.

The topology fixed binary volume is converted into a voxel surface, which is the input for the spherical parameterization. Based on the surface graph, two Laplacian matrixes are constructed for initial parameterization of two polar coordinates, latitude *θ* and longitude *φ*, respectively. After the initial parameterization of latitude and longitude, a non-linear constrained optimization method is used to minimize the area and topology distortions of the surface net in the mapping. Additional file 3: Figure S3 shows how a distinctly labelled protrusion is mapped onto the sphere.

### Shape representation

Let *Ω* be a unit sphere, embedded in *ℝ*^*3*^. The spherical Laplacian *Δ*_*Ω*_ operator on *Ω* satisfies

(30) $$\Delta_{\Omega }Y_{l}^{m}=-l\left(l+1\right)Y_{l}^{m}$$

where $Y_{l}^{m}$ are spherical harmonics (SPHARM). Eq. (30) indicates that SPHARM are the eigenfunctions of *Δ*_*Ω*_ with eigenvalue of *λ*_*l*_ = - *l*(*l* + 1) [30]. Denote the space of square integrable functions with respect to *Ω* as *L*^2^(*Ω*). Define ${\text{Spharm}}_{l}=\mathrm{span}{\left\{Y_{l}^{m}\right\}}_{m=-l}^{l}$ as a subspace of all SPHARM of the same degree *l*, and let ${\text{Spharm}}_{0,\ldots n}={⊕}_{l=0}^{n}{\text{Spharm}}_{l}$. Then it can be rigorously proven that the closure of *Spharm*_0,…*n*_ converges to the space *L*^2^(*Ω*) as *n* → ∞ [31]. Therefore, SPHARM form a complete set of orthonormal basis functions in space *L*^2^(*Ω*), analogue to unit basis vectors. Similarly as vectors can be described by projections onto each axis (scalar product between vectors), expansion coefficients (scalar product between functions) can be used for the description of functions [32]. Any spherical function can be expressed by an infinite series of SPHARM coefficients.

The cell surface *M* defined on *L*^2^(*Ω*) can be expressed as a set of SPHARM coefficients. Each function on the right side of Eq. (29) can be independently decomposed in terms of SPHARM as

(31) $$x\left(\theta ,\varphi \right)=\overset{\infty }{\underset{l=0}{\sum }}\overset{l}{\underset{m=-l}{\sum }}c_{\text{lx}}^{m}Y_{l}^{m}\left(\theta ,\varphi \right)$$

(32) $$y\left(\theta ,\varphi \right)=\overset{\infty }{\underset{l=0}{\sum }}\overset{l}{\underset{m=-l}{\sum }}c_{\text{ly}}^{m}Y_{l}^{m}\left(\theta ,\varphi \right)$$

(33) $$z\left(\theta ,\varphi \right)=\overset{\infty }{\underset{l=0}{\sum }}\overset{l}{\underset{m=-l}{\sum }}c_{\text{lz}}^{m}Y_{l}^{m}\left(\theta ,\varphi \right)$$

This expansion is exact, but errors are introduced when restricting *Spharm*_0,…*n*_ to a finite space with a certain degree of *l*, which is required in numerical implementations. A truncated SPHARM series can be effectively used to fit relatively smooth functions and model surface protrusions and intrusions [14].

In constructing the SPHARM representation of Eqs. (31, 32 and 33), we need to estimate the coefficients $c_{\text{lx}}^{m}$, $c_{\text{ly}}^{m}$, and $c_{\text{lz}}^{m}$. There are three major techniques for estimating those coefficients: The simplest method is to numerically integrate the Fourier coefficients over a high resolution triangle mesh [33], which is computational expensive. The second method is based on the fast Fourier transform [34], in which a predefined regular grid system is required. The most widely used method is based on solving a system of linear equations that minimize the least square problem [35]. The estimated coefficients approximate the full underlying surface, and can be used to represent and reconstruct the surface.

### Shape comparison

The concise representation of cell shape using SPHARM allows for local measurements of cell membrane deformation. However, there are two additional issues to be addressed, one is registration, and the other is re-sampling. In order to obtain more accurate results, it is usually necessary to register two cell surfaces before the comparison can be conducted. The registration process removes arbitrary rigid motions and brings each cell into the same coordinate system. The surface registration step is an optional one depending on the application, which can be performed before the segmentation, directly on the intensities of image voxels, or after the shape representation on the mesh. There are a variety of registration methods available in the literature [10,36].

Normally, the size of the cell mesh varies. It needs to be re-sampled to make the number of vertices in the model for each cell the same. This can be achieved by using the regular mesh in the parameter space for reconstruction. Subsequently, pairwise comparisons between different surface models can be conducted. The correspondence between SPHARM models is implied by the underlying parameterization: two points with the same parameter pair (*θ*, *φ*) on two surfaces define a corresponding pair.

SPHARM allows cell shapes to be well-characterized, both in a static and a dynamic manner [37], and is able to extract a wide range of qualitative and quantitative parameters, such as individual outliers, most commonly occurring shapes, and spatiotemporal patterns of surface deformations. Temporal analysis of SPHARM coefficients has been used to detect major deformation phases, and to identify temporal events of interest such as the formation of blebs as well as patterns of deformation [37]. Fundamental features of cell shape dynamics can be extracted from time series images, which is essential to understand the biophysical mechanisms of cell migration. For the purpose of illustration, we use coefficients to identify five major phases of deformation as shown in Additional file 4: Figure S4, and estimate the temporal local deformations (Additional file 5: Figure S5) of the cell membrane by subtracting cellular models at different time points.

## Results

The experiments were performed using unoptimized Matlab code running on an Intel(R) Core(TM) 2.3 GHz with 6 GB of RAM. In principle, the proposed framework can be used for any 3D cell shape analysis. To demonstrate its performance, ten neutrophil cell sequences were acquired, which were labelled with cell mask orange dye to stain the plasma membrane. The neutrophils are quiescent, then stimulated by application of fMLP which prompts cell spreading and movement. Cell movements are imaged by spinning disk confocal microscopy. The acquisition speed is about 80 ms per slice (4 secs/stack). The size of the stack is 180×283×24, and its scale is 0.16x0.16x0.5 microns per voxel.

### Cell segmentation

We use raw data to test our segmentation method, which are more challenging than deconvolved data. Although deconvolution usually generates smoother data, it is prone to generate artefacts. We compared both the segmentation quality and the time complexity of our method with the random walker [18] and the level set method [38]. For the random walker method, the seeds are detected automatically similarly to the method described in Section 2.3 using the original image without downsampling. We extended the code from [18] to 3D image segmentation. For the level set method, we manually set a region of interest with a right prism and then run the algorithm with 50 iterations using the code from [38]. We use the subsampling rate of 1/4 for all the test image stacks.

**Figure 3:**
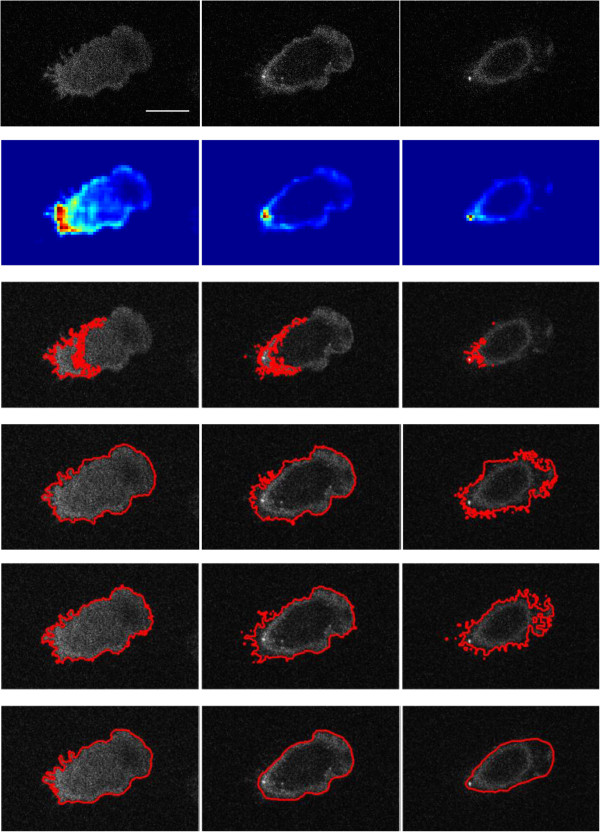
**Comparison of segmentation methods.** Row 1: Original, Row 2: saliency map, Row 3: random walker method, Row 4: level set method, Row 5: our method, Row 6: manually segmented ground truth. The saliency map is used to automatically generate seeds for both the random walker method and our method. Columns 1–3 are the 1st, 4th, and 7th slices of the image stack. The random walker method was unable to obtain proper results, possibly because of noise and inhomogeneous intensity distributions. Our method is comparable to the level set method with respect to dealing with noisy cell boundaries. Regarding efficiency, our method took only 2.62s, while the random walker method and the level set method took 17.82s and 32.09s, respectively. Scale bar: 10 micron.

**Figure 4:**
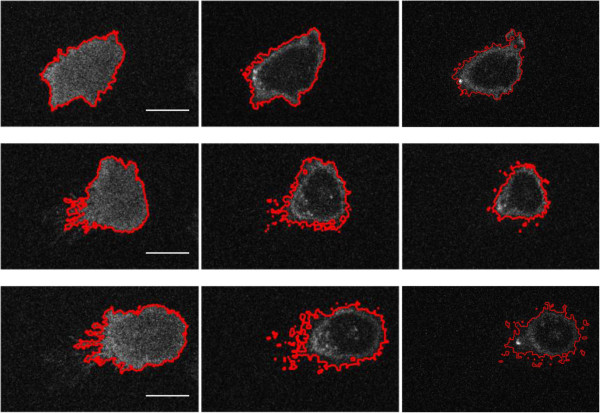
**Segmentation results using our method for three other stacks (Rows 1–3).** Columns 1–3 are the 1st, 4th, and 7th slices of the image stack. It can be seen that currently our method struggles to segment very fine cell processes (retraction fibres). Scale bar: 10 micron.

Figure 3 shows the results for the segmentation of one of the example stacks (Row 1), the saliency map (Row 2), and its ground truth (Row 6). Compared to the raw image, the saliency map is much less noisy, which facilitates thresholding. The random walker method was unable to obtain a proper result (Row 3), possibly because of the noise in the image and the inhomogeneous intensity distribution. We also tried to use the bilateral filter to denoise the slices first before segmentation, but still without much improvement. Our method (Row 5) is comparable to the level set method (Row 4) with respect to dealing with noisy cell boundaries. Regarding efficiency, our method took only 2.62s, while the random walker method and the level set method took 17.82s and 32.09s, respectively. In [17], we manually entered seeds in the middle of the cell image, and similar segmentation results were obtained. Figure 4 shows segmentation results of three other stacks using our method. It can be seen that currently our method struggles to segment very fine cell processes (retraction fibres), which deserve further study.

The global F-measure [39] is calculated to evaluate the segmentation results quantitatively. The ground truth of the whole cell was obtained by using the semi-automatic software ITK-SNAP. Firstly, we imported a real cell image into the software for semi-automatic segmentation. An initial segmentation result was obtained by using the active contour method. The binary segmentation was subjected to manual post-processing to fill holes and remove artefacts. Ten segmented cell stacks and corresponding original images are available on the authors’ website [40]. As the random walker method cannot segment the entire cell, we only compare our approach with the level set method. Our method achieves a better F-measure value of 0.9, when compared with the level set method (0.83). Visual inspection of more than 10 sequences with 230 time points each, showed that the obtained automated segmentation is well within the limits of expected interobserver error.

### Topology fixing

**Figure 5:**
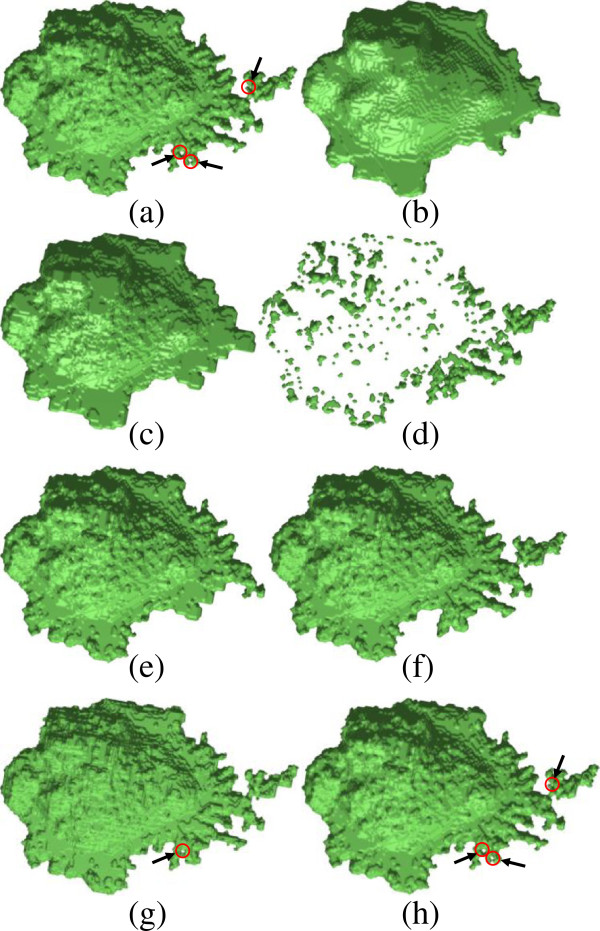
**Topology fixing of an example stack with Euler number -5. (a)** The original binary volume; **(b)** 1.5-sets of the embedding function; **(c)** The binary volume without fine protrusions; **(d)** The detected protrusions; **(e)** The binary volume **(c)** merged with the protrusions that have spherical topology; **(f**-**h)** The final topology fixing results by our method **(f)**, results obtained by SPHARM-PDM **(g)**, and SPHARM-MAT **(h)**. Three holes, each of which was fixed by our method, can be seen from this viewpoint, highlighted by red circles and black arrows. The modification is small, with 0.42% difference between the fixed **(f)** and the original volume **(a)**. While SPHARM-MAT introduced even fewer artefacts (0.06%), and SPHARM-PDM 0.83%, none of the holes were filled by SPHARM-MAT **(h)**, and only two of them were filled by SPHARM-PDM **(g)**.

Seventy-two stacks of neutrophil cell images were randomly chosen and segmented. Among them 64 had no spherical topology. An example stack with ill topology is shown in Figure 5(a), whose Euler number is -5. Three holes that can be seen from this viewpoint are highlighted by red circles. We embed the binary volume into a function with first-order smoothness (see Figure 2 for three slices of the embedding results).

Theoretically, the p value in Eq. (21) can be varied from 1 to ∞. However, the *p* = ∞ case is more complex and computational expensive, and will be a matter of future work. In this paper, we fix the p value to 2 since it has been demonstrated in [18] that the p=2 case achieves better results with less "shrinking bias". Furthermore, the convex quadratic problem of Eq. (25) can be solved easily and efficiently by using a number of convex optimization algorithms. The final result of topology fixing should be similar with respect to the choice of different p values. We also employed the embedding function with second-order smoothness in our experiments, and find that there is not much difference for the current application.

Figure 5(b) shows the binary volume *A* that is 1.5-sets of the embedding function *F*. Our experiments show that the choice of the threshold value of the level is not sensitive in this application. The remaining binary volume *B* without fine protrusions is shown in Figure 5(c). It has spherical topology. Initially, protrusions that possibly violate topology are not detected accurately and contain many false positives as shown in Figure 5(d). However, that will not affect the final result of topology fixing in our application. Nevertheless, it might serve as a good starting point for protrusion detection. The key point here is that the detected protrusions must contain everything that causes a topology violation. Figure 5(e) can be considered as the largest volume we can obtain, which has spherical topology without fixing. In comparison with the original volume of Figure 5(a), it can be seen that only a very small portion of the volume contributes to topology violation. We will leave Figure 5(e) as it is, and only modify a few small protrusions. As demonstrated in Figure 5(f), the modification is almost negligible in the final result with only 0.42% difference between the fixed and the original volume.

Figure 5(g-h) shows the topology fixing results obtained by two open-source softwares, SPHARM-PDM [15] and SPHARM-MAT [16]. The SPHARM-MAT method introduced 0.06% artefacts, which is better than our method (0.42%) and the SPHARM-PDM method (0.83%). However, none of the holes has been filled by the SPHARM-MAT method (Figure 5(h)), while only two of them were filled by the SPHARM-PDM method (Figure 5(g)). We compare the average rates for successful topology fixing of the 64 binary volumes without spherical topology, which are 69%, 4%, and 95%, for SPHARM-MAT, SPHARM-PDM and our proposed method, respectively. The amount of introduced artefacts are 0.21%, 0.96% and 0.94%, respectively.

### Spherical parameterization and shape representation

We have compared the performances of several spherical parameterization methods [10,27-29] and their results of shape representation using SPHARM. The method proposed in [10] and implemented in [41] performed best in our application (results not shown), and will be used in the rest of this paper. The root mean-squared error (RMSE) was utilized to quantify the performance of shape representation results. The RMSE is computed by

$$\text{RMSE}=\sqrt{\frac{\sum_{i=1}^{N}{\left(M\left(p_{i}\right)-\overset{˜}{M}\left(p_{i}\right)\right)}^{2}}{N}}$$

where *M* indicates a true cell surface and $\overset{˜}{M}$ its SPHARM representation, which is evaluated at N sampling points ${\left\{p_{i}\right\}}_{i=1}^{N}$. The average error for all the tested cell images is reported for each method.

An important aspect of measuring a cell’s migratory behaviour is the quantification of local cellular protrusions and retractions. In order to evaluate our method’s ability as regards to what extent small local shape variations can be detected, we artificially synthesized a cell by introducing a controlled local shape deformation using the open source software ITK-SNAP [42]. We imported the ground truth segmentation of a real cell image into the software and manually added a ball to simulate a cellular protrusion by using the paintbrush tool of ITK-SNAP (see Additional file 6: Figure S6).

**Figure 6:**
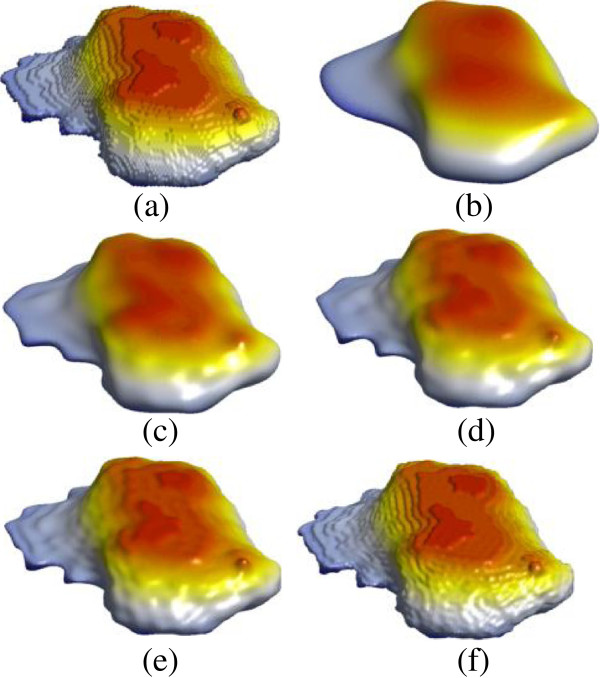
**Synthesized cell where an artificial ball has been inserted into the membrane and its corresponding shape representation with SPHARM. (a)** Synthesized cell, **(b**-**f)** SPHARM degrees of 10, 20, 30, 42, and 78. It is not sufficient to use degrees up to 10. From the models with degrees 20 onwards, we can easily identify the inserted ball and reconstructions improve with increasing SPHARM degrees.

**Figure 7:**
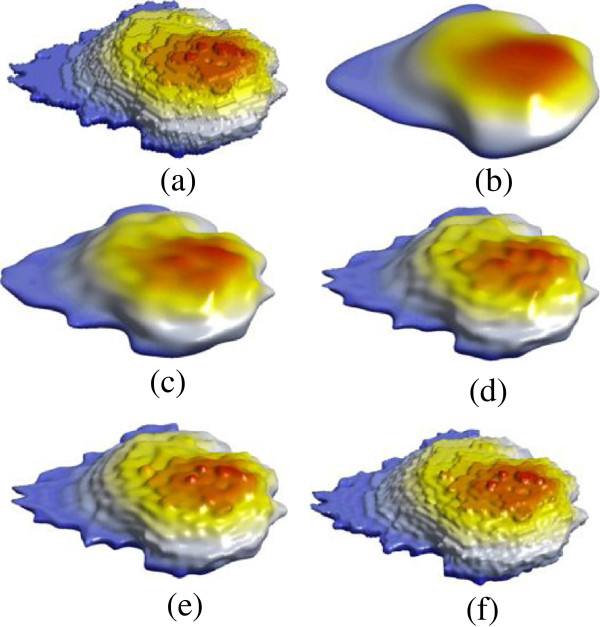
**Original real cell image (a) and its shape representation with SPHARM degrees of 10, 20, 30, 42, and 78 (b-f).** The performance is improved as the degree increases. For better results, we suggest to use the model with degree 42 for a typical cell shape.

From the mesh and its spherical parameterization, the SPHARM description can be computed according to Eqs. (31, 32 and 33), which is essentially a set of coefficients weighting individual spherical harmonic basis functions. This description is then sampled into a triangulated surface via an icosahedron subdivision of the spherical parameterization. We ask how many coefficients are necessary to represent local deformations for a given cell shape. Figure 6 shows SPHARM models of the simulated stack with degrees of 10, 20, 30, and 42, respectively. As we expected, it is not sufficient to use degrees up to 10. From the models with degrees 20 onwards, we can easily identify the artificial ball. However, the models with degrees 20 and 30 are unable to represent the details exhibited in the simulated stack. The SPHARM models of one of the real cell images is shown in Figure 7, with high degrees up to 42. Similarly, the performance is improved as the degree increases. For good quality results we suggest to use models with degree 42 for typical cell shapes, which requires solving 5547 Fourier coefficients in total.

Table 1 reports the average values of RMSE, and the average expansion and reconstruction times for shape representation of cell images using SPHARM. The typical mesh size in the current study is 20426. RMSE decreases from 0.81 to 0.39 as the degree increases from 10 to 42. Theoretically, RMSE can be reduced further by using high enough degrees of SPHARM. However, the running time will be enormous as the number of coefficients to be estimated will increase quadratically. The total running time increase from 4.29 s to 216.12 s when the degrees of SPHARM increase from 10 to 42, where the majority of time is consumed for expansion, i.e. to obtain the coefficients.

**Table 1:** Average RMSE values and average running times for shape representation of cell images using SPHARM

**SPHARM**	**RMSE**	**Expansion time(s)**	**Reconstruction time(s)**	**Total time(s)**
degree10	0.81	3.88	0.41	4.29
degree20	0.53	16.38	2.29	18.67
degree30	0.44	61.43	6.67	68.09
degree42	0.39	199.78	16.35	216.12

## Discussion

In this paper, we develop algorithms for different steps in our framework in a formalized way using Laplacian approaches. Each method can be viewed from the perspective of exploring eigenfunctions of the Laplacian matrix. Although different affinity matrices are used for the first three steps, i.e. the weights defined are application-driven, all of them are symmetric (*A*(*i*, *j*) = *A*(*j*, *i*)), positive preserving (*A*(*i*, *j*) ≥ 0) and positive semi-definite (for all x in *R*^*N*^, *x*^′^*Ax* ≥ 0). In addition all minimize a quadratic distortion measure, naturally leading to the eigenfunctions of Laplacian-type operator [43]. In the fourth step, we use SPHARM for shape representation, which are eigenfunctions of the spherical Laplacian *Δ*_*Ω*_. Therefore, all the techniques used in the first four steps are closely connected. Indeed, it was shown in [44] that the Laplacian of a graph is the discrete analogue of the Laplace-Beltrami operator on manifolds. The spherical Laplacian *Δ*_*Ω*_ is the Laplace–Beltrami operator on the unit sphere *Ω*.

As the affinity matrix for cell segmentation satisfies the conditions of symmetry and pointwise positivity, the pairwise similarities can be interpreted as edge flows in a Markov random walk on the graph [45]. To perform the random walk segmentation, instead of solving the linear system of Eq. (4), one may precompute several eigenvectors of the Laplacian matrix and use this information to produce an approximation of the random walker segmentation algorithm [46]. The approximation can be viewed from the standpoint of distance in the "spectral coordinates" space defined by the weighted generalized eigenvectors.

Furthermore, all the methods used in the four separate steps are closely related to the problem of heat diffusion. The random walk segmentation method can be considered as a diffusion approach [47], where the seeded pixels are treated as the heat sources and the background acts as a sink. After reaching equilibrium the image can be segmented according to the temperature at each pixel.

The top eigenfunctions of Markov matrices (describing local transitions, or affinities in the system) permit a low-dimensional embedding, so that the ordinary Euclidean distance in the embedding space measures intrinsic diffusion metrics on the data [43]. The non-binary embedding approach for the topology fixing can be viewed as a diffusion process subject to the hard constraints. An iterative scheme is used, where the constraints are enforced and diffusion restarted using the new solution.

The initial parameterization of the latitude and longitude are obtained from heat diffusion [10]. Latitude grows smoothly from 0 at the north pole to *π* at the south pole. In a physical analogy, the south pole is heated up to temperature *π*, while the north pole is cooled down to temperature 0. The parameterization results are obtained as the stationary temperature distribution on the heat conducting surface.

Fourier series have long been used for solving diffusion problems analytically. Similarly, the spherical diffusion equation *kΔ*_*Ω*_*u*(*φ*, *θ*, *t*) = ∂ _*t*_*u*(*φ*, *θ*, *t*) can be solved by expressing *u*(*φ*, *θ*, *t*) as SPHARM expansion. SPHARM can be used in isotropic heat diffusion via the Fourier transform on a unit sphere as a means of hierarchical surface representation [34].

Such a perspective can help the reader to better understand the commonalities behind seemingly different techniques. It can also open a door for further improvement of the methods. By exploiting structural similarities of the different approaches, it should be possible to integrate for example the topology fixing step into the cell segmentation process, which will be a future work of our research.

As an illustration of the technique, we have applied it to neutrophil cell shape analysis. The presented methods can be used for modelling arbitrarily shaped but simply connected 3D objects. They are suitable for surface comparison and are able to detect protrusions and invaginations, and quantify their dynamics. Shape plays important roles in many biological processes, such as bimolecular recognition and the problem of protein binding pocket and ligand comparison [32], where the presented methods have potential to extract functional information from protein structures, locally and globally.

One of the limitations of the framework is that it can only be used to represent genus-zero objects, which is true for the cell surface, but not for more complex intracellular structures like the endoplasmatic reticulum. As of now segmentation and spherical parameterization of our method are unable to cope with very thin protrusions.

Currently, we process every frame independently irrespectively of prior knowledge of previous time points, which could be used to initialize segmentation of the current frame. However, since in our application cells moves quite fast, the overlap between the membrane marker at consecutive time points is too low.

## Conclusions

This report presents a framework for 3D+time cell shape analysis, which includes five major steps: cell segmentation, topology fixing, spherical parameterization, shape representation, and shape comparison. We formalize the algorithms for the first four steps using Laplacian approaches. All the methods can be viewed from the perspective of exploring eigenfunctions of the Laplacian matrix, and are closely related to the problem of heat diffusion. We developed a fast random walker method for cell segmentation, which is based on the Laplacian matrix generated from the discrete grid domain and the affinities defined by a Gaussian kernel. It is faster than the other two popular methods with comparable segmentation quality. The novel topology fixing method we proposed is also based on the Laplacian matrix generated from the discrete grid domain, but the affinity matrix contains unit weights. It is able to fix the topology of complex cells with a high success rate while introducing only minor artefacts. The spherical parameterization method we applied is based on the Laplacian matrix generated from the surface graph and unit weights are assigned to each entry of the affinity matrix with some special modifications. For the shape representation, we directly explore the eigensystems of spherical Laplacian without constructing the Laplacian matrix explicitly. The spherical parameterization and the shape representation methods are used for both simulated and real cells, achieving satisfactory results. By analyzing the temporal Fourier spectrum, we are able to identify major deformation phases. The temporal local deformations of the cell membrane can be estimated by subtracting cellular models at different time points. In the future, we will apply our framework for 3D+time neutrophil cell shape analysis to study in detail how dynamic distributions of phospholipids correlate with membrane dynamics.

## Competing interests

The authors declare that they have no competing interests.

## Authors’ contributions

CJD and TB developed and discussed the methods. CJD developed all software. PTH, LRS, and TB conceived and discussed the project. CJD and TB wrote the publication. PTH, LRS provided data for testing the algorithms. All authors have read and approved the final manuscript.

## Supplementary Material

Additional file 1: Figure S1 Segmentation results using our method for a *Dictyostelium* cell labelled with two markers for Lim (mRFP, red) and Coronin (GFP, green). Row 1: original images (courtesy of G. Gerisch, M. Ecke, MPI Biochemistry, Martinsried). Row 2: segmentation results. Columns 1 – 3 are the 11st, 17th, and 23rd slices of the image stack. For segmentation the two channels have been combined into one. Click here for file

Additional file 2: Figure S2 Comparison of edge-stopping functions. A variety of edge-stopping functions have been used such as Lorentz, Gauss, and Tukey’s biweight function. The Lorentz function enhances outliers when compared to the Gauss and the Tukey functions. More robust results can be achieved by Tukey’s biweight function, as it prevents diffusion across edges completely. Click here for file

Additional file 3: Figure S3 Spherical parameterization of a cell surface with protrusions. Nodes within the protrusion are highlighted by red markers **(a)** to demonstrate how a particular region is mapped onto the sphere **(b)**. Click here for file

Additional file 4: Figure S4 Temporal analysis of SPHARM coefficients can be used to distinguish different phases of cellular deformations. **(a)** Temporal SPHARM coefficients of a sequence with *l* = 2, *m* = ±2. **(b**-**f)** Characteristic deformation phases at time points 18, 46, 56, 71, and 116, which differ in cell roundness and symmetry. Click here for file

Additional file 5: Figure S5 Visualising dynamic local deformations for time points 18 to 23 in corresponding Additional file 4**(a**-**f)**. Cell deformations are estimated by subtracting surface reconstructions at different time points. The distance between surfaces is colour coded (black: no deformation, red: protruding regions, blue: retracting regions). Click here for file

Additional file 6: Figure S6 Synthesized cell with local shape deformation (green ball) using the open source software ITK-SNAP. After importing the ground truth segmentation (red) of a real cell image a ball (green) was manually added by using the paintbrush tool to simulate a well defined protrusion and assess the quality of surface reconstructions. Click here for file
