# Supplementary figures and images for: 3D time series analysis of cell shape using Laplacian approaches
Source: BMC Bioinformatics. 2013 Oct 4;14:296. doi: 10.1186/1471-2105-14-296 (PMC3871028; doi:10.1186/1471-2105-14-296)

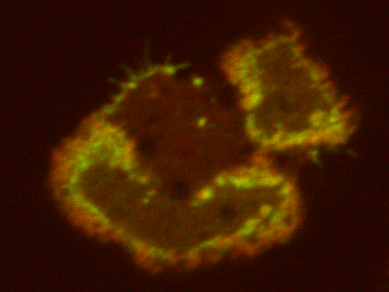

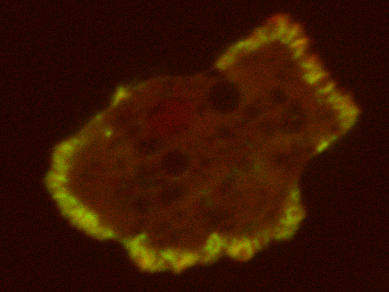

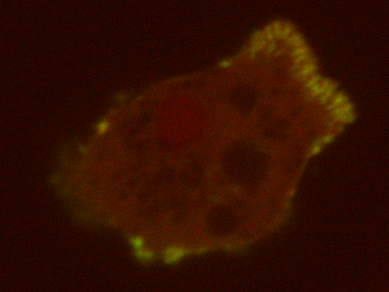


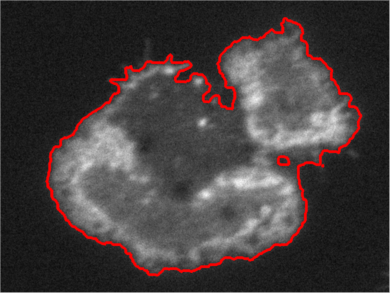

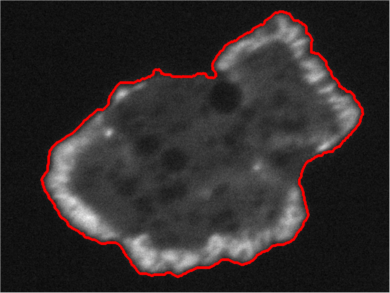

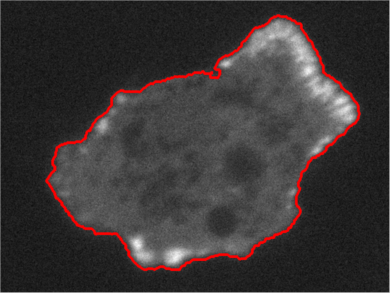

Supplement: Additional file 1: Figure S1 — Segmentation results using our method for a Dictyostelium cell labelled with two markers for Lim (mRFP, red) and Coronin (GFP, green). Row 1: original images (courtesy of G. Gerisch, M. Ecke, MPI Biochemistry, Martinsried). Row 2: segmentation results. Columns 1 – 3 are the 11st, 17th, and 23rd slices of the image stack. For segmentation the two channels have been combined into one. [file 1471-2105-14-296-S1.docx]

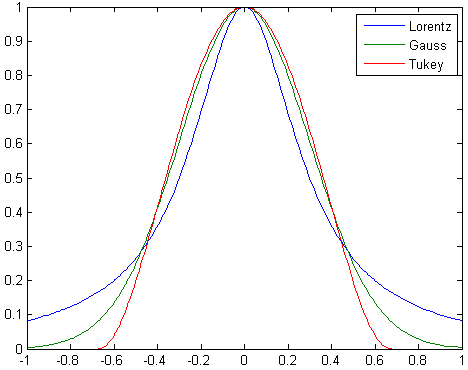

Supplement: Additional file 2: Figure S2 — Comparison of edge-stopping functions. A variety of edge-stopping functions have been used such as Lorentz, Gauss, and Tukey’s biweight function. The Lorentz function enhances outliers when compared to the Gauss and the Tukey functions. More robust results can be achieved by Tukey’s biweight function, as it prevents diffusion across edges completely. [file 1471-2105-14-296-S2.docx]

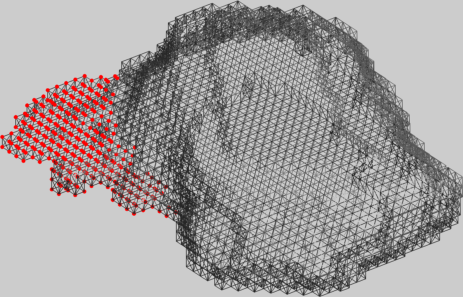

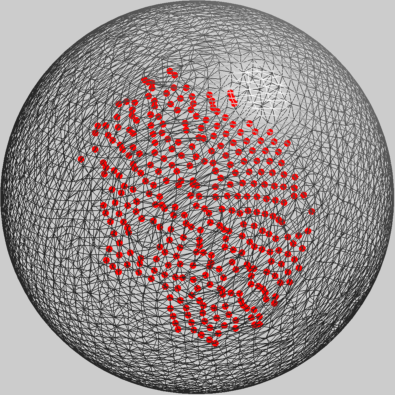


(a)

(b)

Supplement: Additional file 3: Figure S3 — Spherical parameterization of a cell surface with protrusions. Nodes within the protrusion are highlighted by red markers (a) to demonstrate how a particular region is mapped onto the sphere (b). [file 1471-2105-14-296-S3.docx]

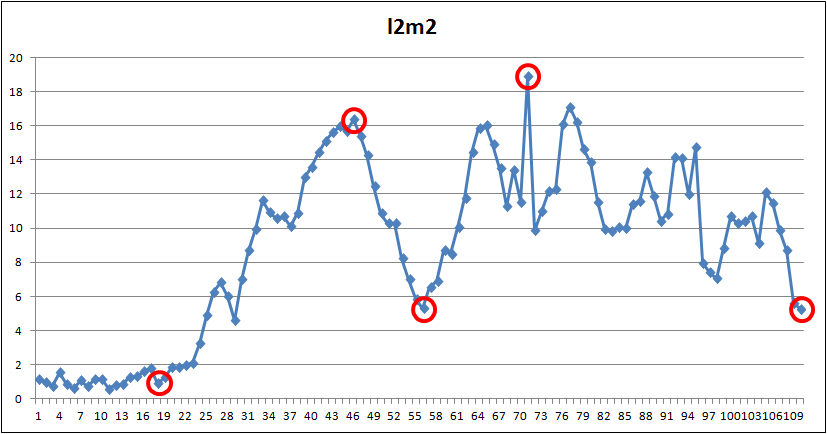


(a)


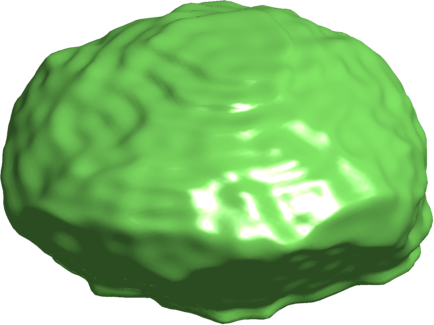

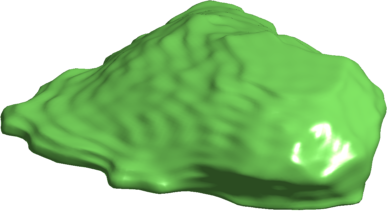

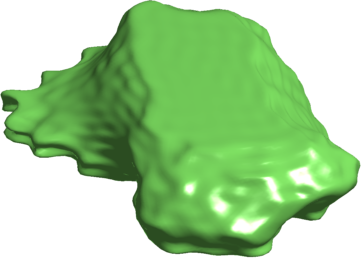


(b)

(c)

(d)


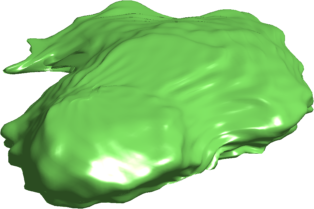

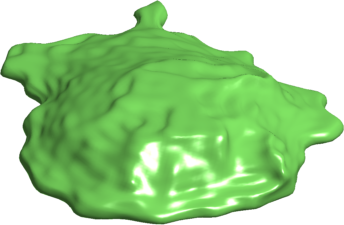


(f)

(e)

Supplement: Additional file 4: Figure S4 — Temporal analysis of SPHARM coefficients can be used to distinguish different phases of cellular deformations. (a) Temporal SPHARM coefficients of a sequence with l = 2, m = ±2. (b-f) Characteristic deformation phases at time points 18, 46, 56, 71, and 116, which differ in cell roundness and symmetry. [file 1471-2105-14-296-S4.docx]

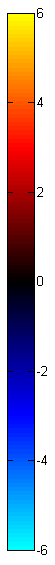


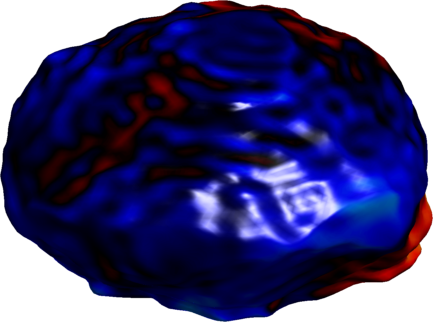

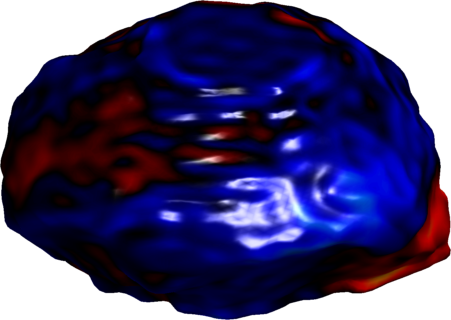


(b)

(a)


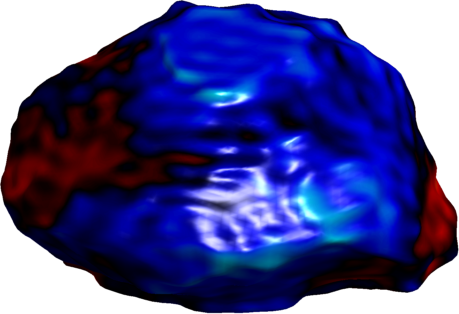

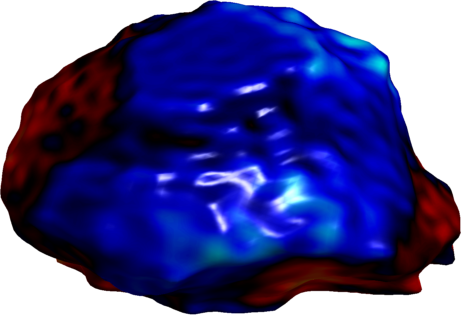


(d)

(c)


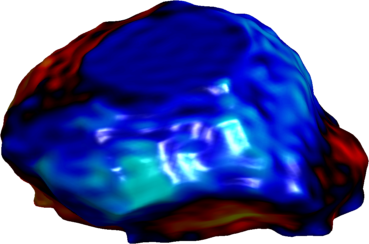

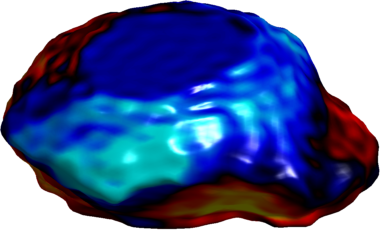


(f)

(e)

Supplement: Additional file 5: Figure S5 — Visualising dynamic local deformations for time points 18 to 23 in corresponding Additional file 4(a-f). Cell deformations are estimated by subtracting surface reconstructions at different time points. The distance between surfaces is colour coded (black: no deformation, red: protruding regions, blue: retracting regions). [file 1471-2105-14-296-S5.docx]

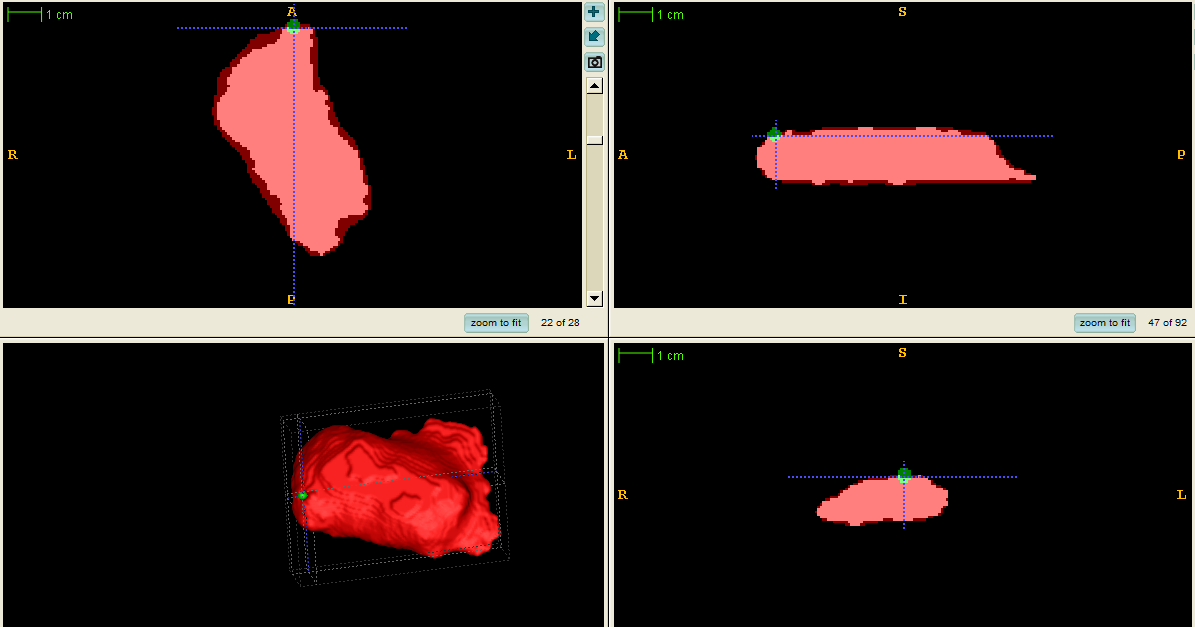

Supplement: Additional file 6: Figure S6 — Synthesized cell with local shape deformation (green ball) using the open source software ITK-SNAP. After importing the ground truth segmentation (red) of a real cell image a ball (green) was manually added by using the paintbrush tool to simulate a well defined protrusion and assess the quality of surface reconstructions. [file 1471-2105-14-296-S6.docx]
